# Investigation and response to rift valley fever outbreak in ruminant livestock from Ngoma District, eastern province of Rwanda, 2024

**DOI:** 10.1016/j.onehlt.2026.101332

**Published:** 2026-01-16

**Authors:** Eugène Niyonzima, Florien Nkurunziza, Jean Damascene Ngaboyimana, Vestine Nyirandahiriwe, Felicien Mvuyekure, Fabrice Ndayisenga, Solange Uwituze, Denyse Mugwaneza, Eric Iradukunda, Vestine Uwitugabiye, Claire Murekatete, Anselme Shyaka

**Affiliations:** a Rwanda Agriculture and Animal Resources Development Board, Veterinary Services, Rubirizi National Veterinary Laboratory, Kigali, Rwanda; b Rwanda Biomedical Center, Public Health Surveillance and Emergencies Preparedness and Response Division, Kigali, Rwanda; c Center for One Health, University of Global Health Equity, Butaro, Rwanda

**Keywords:** Rift Valley fever, Outbreak investigation, One health response, Zoonoses, Rwanda

## Abstract

**Background:**

Rift Valley fever (RVF) is a zoonotic mosquito-borne disease, causing high livestock morbidity and mortality, with potential human spillover. Rwanda has experienced repeated outbreaks, including significant ones in 2018 and 2022. In August 2024, a smaller localized outbreak was reported in Ngoma District, Eastern Province, providing insights into rapid detection, response, and recovery.

**Methods:**

Following confirmation of the index case by RT-PCR, 4062 blood samples were collected through active community and slaughterhouse surveillance. Epidemiological and demographic data were analyzed, and supportive treatment was provided to confirmed cases. A One Health response was implemented, including livestock vaccination, vector control, and coordinated surveillance.

**Results:**

Among sampled animals, 28 (0.69%) tested positive: 14 cattle, 9 goats, and 5 sheep. Sheep showed the highest infection rate (5.4%). Three animals died, yielding a case fatality rate of 10.7%, while 25 recovered after treatment. Positive cases clustered in six sectors near marshlands and the Akagera River. A total of 112,110 animals were vaccinated. No human cases were reported, and the outbreak was contained within 51 days.

**Conclusions:**

Rapid detection, targeted treatment, and mass vaccination, implemented through a multisectoral One Health response, successfully contained the outbreak and prevented human spillover. Sustained surveillance and cross-border coordination remain essential to mitigate future RVF threats.

## 1 Introduction

Rift Valley fever (RVF) is a mosquito-borne zoonosis caused by RVF virus (RVFV), with outbreaks often linked to rainfall-driven increases in vector populations [[1], [2], [3]]. In humans, Rift Valley Fever virus is primarily acquired through direct contact with the blood, tissues, or bodily fluids of infected animals, while mosquito bites serve as a secondary vector route; clinical disease ranges from mild febrile illness to severe outcomes, including hemorrhage, hepatitis, encephalitis, miscarriage, and death [[4], [5], [6], [7]]. In livestock, particularly cattle, goats, and sheep, RVF causes abortion storms, hemorrhage, and high mortality among young animals, resulting in major economic losses [8,9]. Across Africa and the Arabian Peninsula, RVF has repeatedly caused sizable epidemics with transboundary implications [2,10].

Rwanda has reported recurrent cases since 2012, with major national outbreaks in 2018 and 2022 that affected both livestock and humans. In 2022 alone, surveillance recorded 173 confirmed human cases with 22 associated deaths, alongside 1339 livestock cases, 516 livestock deaths and 1254 abortions [4,11]. In response, the government scaled up annual livestock vaccination and strengthened passive surveillance.

Despite these measures, localized outbreaks continue to occur. In August 2024, RVF was detected in Ngoma District (Eastern Province), triggering an immediate One Health response coordinated by veterinary and public health authorities. This report describes the investigation and control of the event, focusing on: (i) the timeline and spatial distribution of cases; (ii) diagnostic testing and positivity by species and surveillance stream; and (iii) interventions (including vaccination, vector control and supportive care) and clinical outcomes among infected animals. We further highlight the role of multisectoral coordination in rapid containment and in preventing human cases.

## 2 Material and methods

### 2.1 Study design

This investigation employed a cross-sectional study design to assess Rift Valley Fever virus (RVFV) occurrence in livestock during the 2024 Ngoma District outbreak. A total of 4062 animals were sampled across all 14 administrative sectors, including those tested through community surveillance and those intended for slaughtering. In this context, community surveillance refers to active sampling of livestock directly on farms within the outbreak-affected sectors, including both apparently healthy animals and those showing clinical signs, capturing routine herd conditions.

Animals were purposively selected in collaboration with local veterinary officers and farmers; no formal exclusion criteria were applied. In farms where animals with suspected clinical signs were identified, all animals were tested, and if positive, adjacent farms were subsequently sampled. Additionally, all animals presented for slaughter were systematically tested.

Classical RVF-associated risk variables, including history of abortion, grazing system, communal contacts at water points, and acaricide application, were not assessed due to logistical and resource constraints, as the primary objective was to determine viral presence and support rapid outbreak response. Metadata collected for each sampled animal included species, sex, age, vaccination status, observed clinical signs, production system, and sector of origin. These data were primarily obtained through farmer recall, supported where available by farm records and veterinary verification, and were intended to enable stratified analyses and account for potential confounding factors.

### 2.2 Study area

This outbreak happened in Ngoma District, Eastern Province of Rwanda which covers 867.7 km^2^ and comprises 14 administrative sectors (a sector is third level of administrative subdivision in Rwanda's administrative structure below the district and province). According to the 2022 census, the district has a population of 404,048, with 91% residing in rural areas [12]. Ngoma borders Burundi to the south and lies approximately 60 km from the Rusumo border with Tanzania, creating potential for cross-border livestock movement. The district is characterized by low hills, marshlands, and lakes at altitudes of 1400–1700 m. Its bimodal rainfall pattern and widespread livestock farming (cattle, goats, sheep) make it a high-risk zone for vector-borne diseases.

Ngoma has experienced multiple RVF outbreaks in the past, underscoring its status as a high-risk area.

### 2.3 Sample collection

Between 19 August and 3 October 2024, a cross-sectional sampling was conducted across all 14 administrative sectors of Ngoma District to capture a geographically representative distribution of animals. Blood samples were collected from animals raised under family farm production systems within the community and from animals presented for slaughter at local abattoirs, thereby including both on-farm and market-bound populations. Selection of animals at the community level was carried out in collaboration with local veterinary officers and farmers, targeting apparently healthy animals as well as those showing clinical signs, in order to reflect routine herd conditions. At abattoirs, animals were sampled consecutively on slaughter days to minimize selection bias.

Blood was collected aseptically *via* jugular venipuncture into 4 mL EDTA tubes. Each sample was uniquely labeled with the animal ear tag number and date of collection to ensure traceability. Immediately after collection, samples were placed in cool boxes containing ice packs and maintained under cold-chain conditions during transport. All samples were transported on the same day or within 24 h to the National Veterinary Laboratory in Rubirizi, Kigali, for laboratory analysis.

To support assessment of sample representativeness and facilitate stratified analyses, metadata were systematically collected at the time of sampling. These included animal species, sex, age category, vaccination status, observed clinical signs, production system, and precise farm or origin sector. Data were initially recorded on standardized paper-based forms and subsequently entered into a Microsoft Excel database for data cleaning, management, and analysis. Blood samples and associated metadata were collected by trained veterinary officers and field technicians, all of whom had prior experience in aseptic blood collection and were briefed on biosafety and biosecurity measures before field deployment.

### 2.4 Laboratory analysis

Samples were pooled in groups of 10 and centrifuged at 4000 rpm for 5 min. Plasma aliquots (200 μL) were processed using the Maxwell® RSC Viral Total Nucleic Acid Purification Kit (Promega, USA), with RNA eluted in 50 μL nuclease-free water and stored at −20 °C.

RVFV RNA was amplified using the RealStar® RVF Virus RT-PCR Kit 1.0 (Altona Diagnostics, Germany) on a Bio-Rad CFX96™ system, following the manufacturer's protocol. Cycling conditions were reverse transcription at 55 °C for 20 min, denaturation at 95 °C for 2 min, followed by 45 amplification cycles of 95 °C for 15 s, 55 °C for 45 s (with fluorescence acquisition), and 72 °C for 15 s. Positive pools were retested individually to identify infected samples.

### 2.5 Treatment protocol

All RVFV-positive animals identified during the outbreak received supportive veterinary care under sanitary mandate as per the Rwandan standard management protocol for RVFV. Treatment included Vitamin K, an injection of a non-steroidal anti-inflammatory drug (NSAID) (pyrazolone, Castralgin®), penistreptomycin, and phenylbutazone, along with Permapy® acaricide for ectoparasite control. Doses were administered according to the manufacturers' guidance. Clinical recovery was monitored through daily follow-up until resolution or death.

### 2.6 Human case surveillance

Human surveillance was conducted alongside animal outbreak investigations to identify potential zoonotic transmission. Passive surveillance data were obtained from the national health reporting system, capturing suspected and confirmed cases presenting to health facilities in affected and adjacent districts during the outbreak period. Case identification followed national case definitions, incorporating clinical criteria, epidemiological linkage to affected animals or outbreak areas, and laboratory confirmation where indicated. Health facilities in high-risk zones were sensitized to enhance detection of compatible cases.

### 2.7 Statistical analysis

Data were analyzed in R version 4.3.1 [13]. Descriptive statistics summarized RVF test results by species, sex, age and sector. Associations were assessed using Fisher's Exact Test (2 × 2 tables) and Chi-squared or Fisher's with Monte Carlo simulation (larger tables). Odds ratios with 95% confidence intervals were calculated for binary comparisons. Logistic regression evaluated species and sex as predictors of RVF positivity, with model fit assessed using the Hosmer-Lemeshow test.

Given the small number of positive cases and multiple zero-event sectors, multilevel or sector-adjusted regression models were not fitted due to risks of instability; sector-level clustering was instead assessed using stratified analyses and exact or simulated tests.

### 2.8 Ethical considerations

The animal data presented in this study were collected as part of routine surveillance activities conducted under the mandate of the national veterinary services, which authorizes data reporting and dissemination for outbreak investigation purposes. Ethical clearance for publication of anonymized animal data was not required, as no identifiable information related to animal owners or locations was included. All procedures adhered to national veterinary guidelines and standard biosafety protocols to ensure animal welfare and data integrity. Additionally, human data were monitored through passive surveillance in accordance with national public health protocols, without direct human sampling.

## 3 Results

### 3.1 Characteristics of sampled animals

**Fig. 1:**
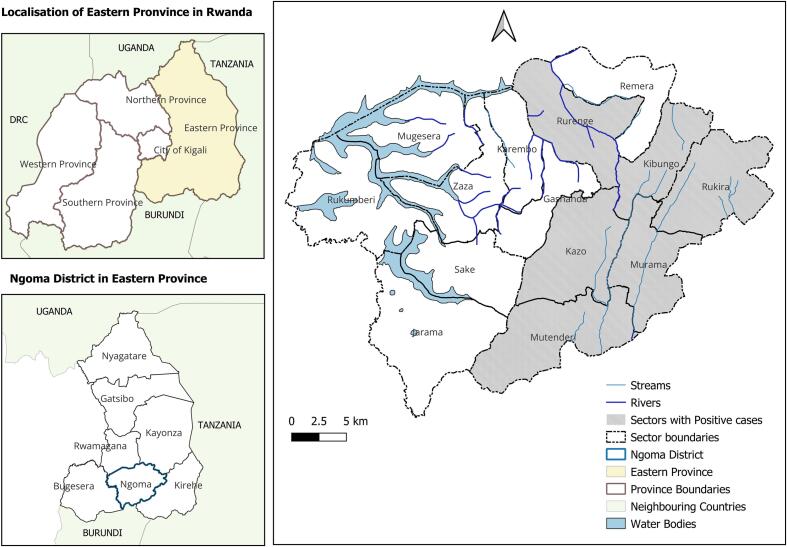
Map of Ngoma district highlighting sectors with positive cases (QGIS Ver. 3.28 - CC-BY license 4.0.). The layers are freely accessible from https://www.diva-gis.org/datadown and can be shared under CC-BY license 4.0.

Between 19 August and 3 October 2024, 4062 livestock blood samples were collected across all 14 sectors of Ngoma District through community and slaughterhouse surveillance. Species composition was 2187 goats (53.8%), 1780 cattle (43.8%), and 95 sheep (2.3%); most animals were female (66.3%). Sectors with the highest sampling contributions included Kibungo (18.1%), Mutenderi (14.3%), and Rurenge (11.8%). Characteristics of the sampled population are summarized in Table 1, while the geographical distribution of sectors with positive cases is presented in Fig. 1.

**Table 1:** Characteristics of sampled animals during the RVF investigation, Ngoma District (*N* = 4062).

Variable	Category	n	%
Species	Goat	2187	53.8
	Cattle	1780	43.8
	Sheep	95	2.3
Sex	Female	2695	66.3
	Male	1367	33.7
Sector of origin	Kibungo	736	18.1
	Mutenderi	582	14.3
	Rurenge	480	11.8
	Kazo	279	6.9
	Zaza	258	6.4
	Remera	252	6.2
	Rukira	241	5.9
	Murama	240	5.9
	Rukumberi	236	5.8
	Mugesera	226	5.6
	Gashanda	180	4.4
	Sake	157	3.9
	Karembo	133	3.3
	Jarama	62	1.5
Sampling venue	Community surveillance	2405	59.2
	Slaughterhouse	1657	40.8
RVF test result	Negative	4034	99.3
	Positive	28	0.7
Treatment among positives	Yes	25	89.3
	No	3	10.7
Recovery among treated	Yes	25	89.3
	No	3	10.7
Mean age (months)	–	22.2	(SD 17.5)
Mean recovery (days)	–	11.7	(SD 8.3)

Baseline distributions of species, sex, sector of origin, surveillance stream (community vs slaughterhouse), RVF status, treatment given, recovery outcome, and summary statistics (mean age, mean recovery time).

### 3.2 RVFV positivity by species and surveillance stream

Overall, 28/4062 (0.69%) animals were RT-PCR positive: 14/1770 cattle (0.79%), 9/2199 goats (0.41%), and 5/93 sheep (5.38%). Positivity was 0.66% at slaughterhouses (11/1657) and 0.70% in community surveillance (17/2405).

**Fig. 2:**
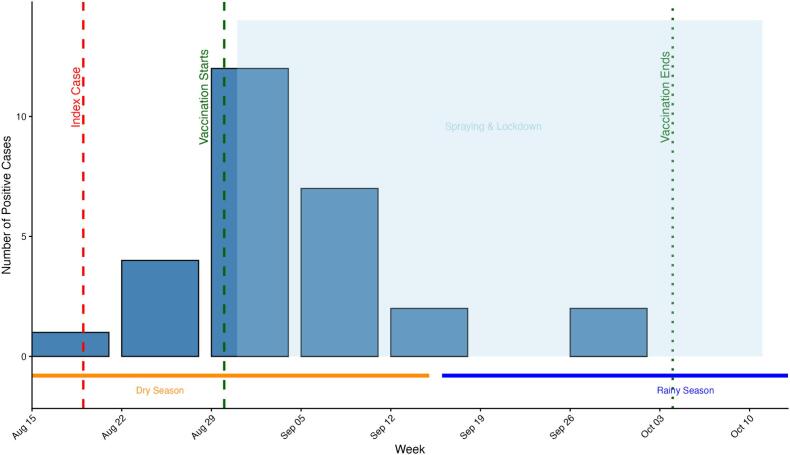
Epidemic curve of RT-PCR–confirmed RVF cases in livestock, Ngoma District, 19 August–3 October 2024. Weekly confirmed cases with timing of interventions (vaccination, spraying, movement restrictions) and seasonal context (dry vs. rainy). Cases peaked in early September and declined rapidly thereafter.

The epidemic curve (Fig. 2) shows a rapid rise after the index case in late August, peaking in early September, followed by a decline after vaccination and vector control. No new positive cases were detected after early October.

Confirmed positive cases were found in 6 of the 14 sectors: Mutenderi (*n* = 11), Kazo (*n* = 5), Murama (*n* = 4), Kibungo (*n* = 3), Rurenge (n = 3), and Rukira (*n* = 2). These sectors are located in wetlands or along the Akagera River, ecological settings favorable for mosquito breeding.

### 3.3 Characteristics of RVF-positive animals, outcomes, and statistical associations

Most positive cases were 12–30 months old, with the largest share in the 12–18-month group. Females comprised 22/28 (78.6%) positives.

All 28 positives received supportive care; 25 recovered and 3 died (overall the case fatality rate, CFR = 10.7%). The Recovery was 86% in cattle, 89% in goats, and 100% in sheep.

Contingency analyses revealed significant associations between RVF positivity and both species and sector. Fisher's Exact Test showed a strong association between species and RVF status (*p* = 0.00018). Sector was also significantly associated with RVF positivity based on Fisher's Exact Test with Monte Carlo simulation (*p* = 0.0072), consistent with the Pearson's Chi-squared test with simulated *p*-value (X^2^ = 31.95, *p* = 0.0053). No significant association was found between sex (*p* = 0.228) or surveillance stream (p = ns).

In the logistic regression model adjusting for species, sex, and age group, sheep had significantly higher odds of RVF positivity compared to cattle (OR = 6.73, 95% CI: 2.13–18.08; *p* < 0.001). Neither goats (OR = 0.54, 95% CI: 0.22–1.24; *p* = 0.15) nor male sex (OR = 0.62, 95% CI: 0.23–1.47; *p* = 0.31) were significantly associated with RVF status. Age group was also not a significant predictor: animals <2 years had higher odds of positivity than those ≥2 years (0.87% *vs* 0.50%), but this difference did not reach significance (adjusted OR = 1.73, 95% CI: 0.79–3.85; *p* = 0.29). The overall model demonstrated good fit (Hosmer–Lemeshow X^2^ = 1.51, df = 2, *p* = 0.47).

Detailed results are summarized in Table 2.

**Table 2:** RVF-positive animals by species, sex, age group, and sector, with outcomes and statistical associations.

Variable	Category	Positives n/N (%)	Recovery n (%)	CFR (%)	p-value (bivariate)	Adjusted OR (95% CI)
Species	Cattle (ref)	14/1770 (0.79)	12 (86)	14.3	–	1
	Goat	9/2199 (0.41)	8 (89)	11.1	0.15	0.54 (0.22–1.24)
	Sheep	5/93 (5.38)	5 (100)	0	0.00018*	6.73 (2.13–18.08)*
Sex	Female (ref)	22/2695 (0.82)	20 (91)	9.1	–	1
	Male	6/1367 (0.44)	5 (83)	16.7	0.228	0.62 (0.23–1.47)
Age group	<2 years	18/2077 (0.87)	–	–	0.29 (ns)	1.73 (0.79–3.85)
	≥2 years (ref)	10/1985 (0.50)	–	–	–	1
Surveillance stream	Community	17/2405 (0.70)	–	–	ns	–
	Slaughterhouse	11/1657 (0.66)	–	–	–	–
Sector	Mutenderi	11/582 (1.89)	–	–	0.0072*	–
	Kazo	5/279 (1.79)	–	–		–
	Murama	4/240 (1.67)	–	–		–
	Kibungo	3/736 (0.41)	–	–		–
	Rurenge	3/480 (0.63)	–	–		–
	Rukira	2/241 (0.83)	–	–		–
	Zaza	0/258 (0.00)	–	–		–
	Remera	0/252 (0.00)	–	–		–
	Rukumberi	0/236 (0.00)	–	–		–
	Mugesera	0/226 (0.00)	–	–		–
	Gashanda	0/180 (0.00)	–	–		–
	Sake	0/157 (0.00)	–	–		–
	Karembo	0/133 (0.00)	–	–		–
	Jarama	0/62 (0.00)	–	–		–

Positives shown as n/N (%). Recovery and CFR within each stratum. Bivariate p-values from Fisher's Exact (2 × 2) or Chi-square with Monte Carlo (larger tables). Logistic regression adjusted for species and sex. Ref = reference category.

### 3.4 Recovery time comparisons

Mean recovery time (Table 3) differed significantly between species: 13.6 ± 12.4 days in cattle, 18.1 ± 7.1 days in goats, and 19.0 ± 0.0 days in sheep (ANOVA *p* < 0.05). Goats and sheep tended to recover more slowly than cattle.

**Table 3:** Mean recovery time among RVF-positive animals by species, Ngoma District, 2024.

Species	Positives (n)	Recovered n (%)	Deaths n (%)	CFR (%)	Mean recovery (days ± SD)
Cattle	14	12 (86)	2 (14)	14.3	13.6 ± 12.4
Goat	9	8 (89)	1 (11)	11.1	18.1 ± 7.1*
Sheep	5	5 (100)	0 (0)	0.0	19.0 ± 0.0*
Total	28	25 (89)	3 (11)	10.7	–

Species-specific sample size (n), mean recovery days (± SD), and one-way ANOVA p-value for between-group differences.

Note: One-way ANOVA showed significant between-species differences in recovery time (p < 0.05). Asterisks () denote means significantly different from cattle (reference group). *.

### 3.5 Outbreak response and vaccination

Following confirmation of the index case, multi-sectoral response teams were rapidly deployed to the affected areas. These included personnel from the Rwanda Agriculture and Animal Resources Development Board (RAB), the Rwanda Biomedical Centre (RBC), local authorities, animal resources officers, and private veterinary service providers under sanitary mandate. In total, 112,110 animals were vaccinated across the district. The outbreak was successfully contained within 51 days, with daily follow-up of at-risk animals and field teams. No new cases were reported after early October 2024.

Contingency analyses revealed significant associations between RVF positivity and both species and sector. Fisher's Exact Test showed a significant association between animal species and RVF status (*p* = 0.00018). Sector was also significantly associated with RVF positivity based on Fisher's Exact Test with Monte Carlo simulation (*p* = 0.0072), consistent with the Pearson's Chi-squared test with simulated *p*-value (X^2^ = 31.95, *p* = 0.0053). No significant association was found between sex and RVF status (*p* = 0.228).

In the logistic regression model adjusting for species and sex, sheep had significantly higher odds of RVF positivity compared to the reference species (OR = 6.73, 95% CI: 2.13–18.08, *p* < 0.001). Neither goats (OR = 0.54, 95% CI: 0.22–1.24, *p* = 0.15) nor male sex (OR = 0.62, 95% CI: 0.23–1.47, *p* = 0.31) were significantly associated with RVF status. The model demonstrated good fit as assessed by the Hosmer-Lemeshow test (X^2^ = 1.51, df = 2, *p* = 0.47).

## 4 Discussion

This outbreak investigation in Ngoma District identified 28 positive animals (0.69%) out of more than 4000 samples tested, with no human cases detected. Sheep had significantly higher odds of positivity compared to cattle, while goats and sex were not significant predictors. Age was not statistically associated with infection, although animals <2 years had a numerically higher positivity rate than older animals. Positives clustered in six sectors along marshlands and the Akagera River, and the outbreak was contained within 51 days following rapid vaccination and spraying of over 112,000 animals. The overall case fatality rate was 10.7%, and recovery time differed significantly between species, with cattle recovering faster than goats and sheep. These findings highlight the importance of early detection, risk-based interventions, and multisectoral response mechanisms in preventing wider RVF spread.

Our results reinforce the well-documented susceptibility of sheep to RVF, which has been observed in multiple outbreaks across East Africa [10,[14], [15], [16]]. In contrast, goats and cattle are typically less severely affected.

In our data, age was not a statistically significant predictor of RVF status, although animals <2 years showed a higher proportion of positives compared to older animals. This difference did not reach significance, likely due to the limited number of positives. Nevertheless, other studies have reported increased vulnerability among younger animals, attributed to immunological naiveté and incomplete vaccination coverage [17,18].

The clustering of cases in marshland and riverine ecosystems is consistent with studies linking RVFV amplification to vector breeding habitats and seasonal rainfall patterns [19]. The relatively low overall positivity (0.69%) contrasts with larger-scale outbreaks in Rwanda in 2018 and 2022, which were more widespread and resulted in higher case burdens [20].

It is also possible that RVFV circulates at a low level in endemic areas between major epidemics, leading to occasional small, non-patterned outbreaks at the margins of larger epidemic cycles as highlighted by various studies in Rwanda and neighboring countries [6,[21], [22], [23]]. Such localized events highlight the importance of continuous surveillance, as they may precede or follow wider transmission events depending on ecological and climatic drivers.

Supportive treatment achieved high recovery (89%), with a case fatality rate lower than reported in some previous RVF outbreaks [4]. The treatment protocol, which included vitamin K supplementation, anti-inflammatory and analgesic support, broad-spectrum antibiotics, and acaricide application, was primarily aimed at symptomatic relief, prevention of hemorrhagic complications, reducing the risk of secondary bacterial infections, and limiting vector exposure, consistent with standard supportive care for RVF in livestock as reported before [4]. The observed interspecies differences in recovery time (cattle faster than goats and sheep) may reflect variation in pathogenesis, host resilience, or veterinary care-seeking behavior [24,25]. Although ANOVA confirmed differences in recovery time, the clinical implications may be modest given the small sample size.

The absence of human cases underscores the effectiveness of rapid animal-side control in preventing spillover. Previous studies have shown that human infections are closely tied to animal outbreaks, with risk elevated by handling of infected carcasses or exposure to vectors in outbreak zones [26]. The close coordination between RAB, RBC, local authorities, and sanitary-mandated veterinarians illustrates Rwanda's strengthening capacity for multisectoral outbreak management. This model highlights the need for: Pre-season vaccination campaigns, with emphasis on sheep and younger animals. Integrated animal - human surveillance, ensuring that confirmation of livestock outbreaks triggers enhanced human surveillance in affected communities. Ecological risk mapping, especially along marshlands and river corridors, to guide proactive interventions. Cross-border coordination with Burundi and Tanzania to prevent re-introduction, given the porous livestock movement across frontiers.

A major strength of this study was the large, district-wide sampling effort across all 14 sectors, providing both community and slaughterhouse data as well as the use of molecular confirmation (RT-PCR) to minimize false positive that could results from vaccination. Lastly, the statistical analyses also accounted for sparse data by applying Fisher's Exact Test with Monte Carlo simulation and logistic regression.

However, the study had limitations. The relatively small number of positives may have reduced the power to detect associations with some independent variables such as age. Entomological data were not collected, limiting our ability to directly link outbreak dynamics to vector abundance. Human surveillance in outbreak sectors was limited to passive reporting, so asymptomatic or mild infections could have been missed. Finally, pooled testing may have slightly reduced sensitivity, though this was mitigated by re-testing positive pools individually.

The implementation of a multi-sectoral One Health response to the Rift Valley Fever outbreak posed several operational challenges. Coordination across veterinary, public health, and local administrative teams required sustained communication to align priorities. Limited resources, including personnel, diagnostic kits, and transport, constrained timely field operations, while variations in stakeholder engagement and logistical challenges in remote areas affected the uniform implementation of sampling and awareness activities. These experiences highlight that effective One Health responses rely not only on technical planning but also on strategic coordination and resource mobilization, offering practical lessons for colleagues managing outbreaks in similar settings.

## 5 Conclusion

The 2024 RVF outbreak in Ngoma District was rapidly detected and contained through coordinated vaccination, vector control, and supportive treatment, preventing human spillover. Findings emphasize the disproportionate susceptibility of sheep, the role of ecologically receptive wetlands, and the value of early multisectoral action. Sustained risk-based vaccination, ecological monitoring, and integrated surveillance will be critical to mitigate the risk of future RVF resurgence in Rwanda and similar endemic settings [27,28].

## Funding

None.

## Ethical approval

Not required.

## CRediT authorship contribution statement

**Eugène Niyonzima:** Writing – review & editing, Writing – original draft, Visualization, Validation, Supervision, Formal analysis, Data curation, Conceptualization. **Florien Nkurunziza:** Methodology, Investigation, Formal analysis. **Jean Damascene Ngaboyimana:** Methodology, Investigation, Formal analysis. **Vestine Nyirandahiriwe:** Methodology, Investigation, Formal analysis. **Felicien Mvuyekure:** Methodology, Investigation, Formal analysis. **Fabrice Ndayisenga:** Writing – review & editing, Writing – original draft, Funding acquisition, Formal analysis, Data curation, Conceptualization. **Solange Uwituze:** Writing – review & editing, Writing – original draft, Funding acquisition, Conceptualization. **Denyse Mugwaneza:** Methodology, Investigation, Formal analysis. **Eric Iradukunda:** Methodology, Investigation, Formal analysis. **Vestine Uwitugabiye:** Methodology, Investigation, Formal analysis. **Claire Murekatete:** Methodology, Investigation, Formal analysis. **Anselme Shyaka:** Writing – review & editing, Writing – original draft, Validation, Supervision, Formal analysis, Data curation, Conceptualization.

## Declaration of competing interest

The authors declare that they have no known competing financial interests or personal relationships that could have appeared to influence the work reported in this paper.

## Data Availability

Data will be made available on request.
